# Isolating error-based and reward-based learning in a real-world task via gradual perturbations in embodied VR

**DOI:** 10.1016/j.isci.2026.117523

**Published:** 2026-09-15

**Authors:** Federico Nardi, Aaruni Arora, A. Aldo Faisal, Shlomi Haar

**Affiliations:** 1 UKRI Centre for Doctoral Training in AI for Healthcare, Imperial College London, London, UK; 2 Care Research & Technology Centre, UK Dementia Research Institute, London, UK; 3 Department of Computing, Imperial College London, London, UK; 4 Department of Bioengineering, Imperial College London, London, UK; 5 Chair in Digital Health & Data Science, University of Bayreuth, Bayreuth, Germany; 6 Department of Brain Sciences, Imperial College London, London, UK; 7 School of Psychology, University of Surrey, Guildford, UK

**Keywords:** motor learning, error-based learning, reward-based learning, visuomotor rotation, gradual perturbation, embodied virtual reality, mobile EEG, post-movement beta rebound, real-world neuroscience, pool billiards

## Abstract

Error-based and reward-based mechanisms act together in real-world motor learning. Laboratory tasks separate them by manipulating feedback, but we previously showed that in a real-world task the correction of a large error is rewarding in itself, and engages reward-based learning even without a reward signal. Here, we asked whether they separate when errors are kept small. We used embodied virtual reality of pool billiards, in which the visual scene is aligned with a physical table, and rotated the cue ball’s seen trajectory gradually while haptics and proprioception stayed unchanged. Thirty-two participants played two sessions, learning the same rotation with error-only feedback in one and reward-only feedback in the other. Their exploration after failed shots and their post-movement beta rebound over the motor cortex differed between sessions. Gradual perturbations can therefore isolate the two mechanisms in a real-world task, and open a way to target them in rehabilitation and skill training.

## Highlights

• Gradual visual perturbations in embodied VR enable isolating motor learning mechanisms
• Error and reward feedback reach the same learning through different strategies
• The small errors prevent error corrections from recruiting reward-based learning
• Post-movement beta rebound declines with reward but not with error feedback

## Graphical abstract

**Figure:**
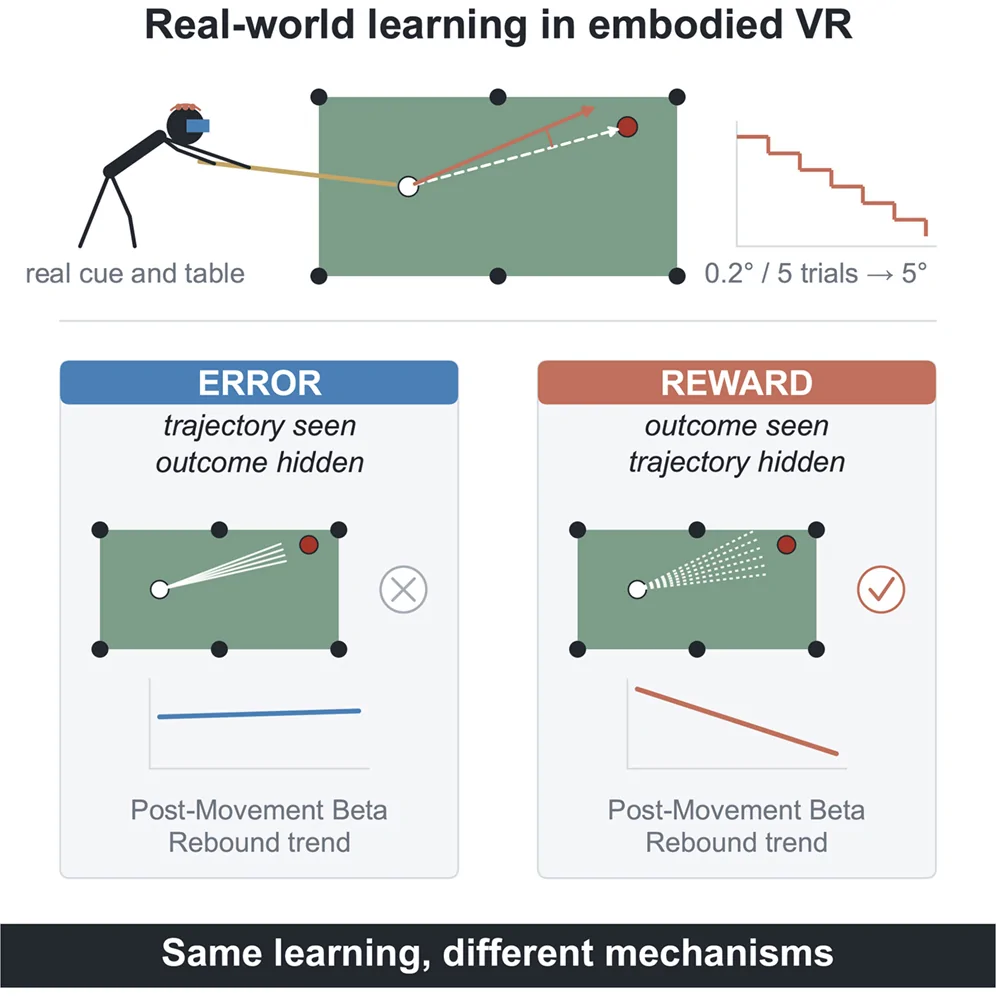

## Teaser

Health sciences; Medicine; Medical specialty; Neurology

## Introduction

Human motor learning is a dynamic process that involves updating internal models of the body and the environment to minimize errors and optimize performance. It relies on a complex interplay of neural mechanisms, including error-based learning and reinforcement learning, alongside predictive processes that anticipate action outcomes. *Error*-based learning adjusts motor commands to reduce the discrepancy between predicted and actual movement outcomes,^1^ and is thought to rely on adaptation processes within the cerebellum^2^^,^^3^; while *reward*-based reinforcement learning optimizes movement behaviors based on reward signals,^4^ and is linked to reward pathways in the basal ganglia and the release of dopamine, which plays a critical role in encoding the value of specific motor actions.^5^^,^^6^^,^^7^

These two distinct learning mechanisms are often investigated in visuomotor rotation paradigms, a well-established reductionistic lab-based framework in which the visual feedback of a movement is rotated relative to the actual trajectory and motion of the hand.^8^^,^^9^^,^^10^^,^^11^ Within these paradigms, two commonly studied perturbation schedules—*abrupt* and *gradual*—are employed to differentially engage the underlying learning systems.^12^^,^^13^ These schedules vary in how prediction errors are introduced, which, in turn, influences how internal models are updated over time and which neural systems are predominantly recruited.

In the reductionist, abrupt perturbation paradigms, a large rotation (e.g., 30°–60°) is suddenly introduced from one trial to the next and kept constant throughout the learning process, creating immediate and significant sensory prediction errors. This evokes strong participation of error-based learning mechanisms, which is associated with rapid changes in motor output. Behaviorally, abrupt adaptation typically follows a double-exponential decay in performance error, consistent with models that involve interacting fast and slow learning processes.^14^ In contrast, gradual perturbation introduces the same total rotation as abrupt, but does so incrementally, increasing the perturbation over multiple trials (e.g., +1° every 5 trials), often without the participant’s explicit awareness.

Moving from reductionist tasks to the real world, recent studies have investigated how the brain learns movements in realistic scenarios, providing valuable insights into motor learning and adaptation.^15^^,^^16^^,^^17^ However, studying movements in the real world presents challenges due to the difficulty in controlling feedback types and learning processes compared to controlled lab settings.

To overcome these limitations, we created an embodied virtual reality (EVR) framework of the billiards game.^18^^,^^19^ Our approach allows us to manipulate the visual feedback while preserving the natural characteristics of the task, including all non-visual sensory feedback. Our system combines a physical pool table, cue stick, and balls with a veridically aligned virtual 3D environment where participants’ motions are captured and tracked in real time using computer vision and motion capture technology. Participants interact with the same objects in both physical and virtual realities (VRs) (they see in VR the pool cue stick they hold physically in their hand, and receive the real-world proprioceptive feedback of hitting the cue ball), enabling us to manipulate the visual environment while they execute realistic movements with comprehensive real non-visual sensory feedback.

In our previous work with this EVR setup, we found that while introducing an abrupt perturbation with error-only or reward-only binary feedback, there were differences in the learning process; however, participants displayed reward-like learning patterns even while receiving error-only feedback.^20^^,^^21^ This suggests that while experiencing large errors, the error correction process is rewarding enough to engage the reward-based learning mechanism.

In this work we used a gradual perturbation strategy, constraining the learning across conditions to be similar in each set of trials with equivalent perturbation magnitudes. Thus, we enabled a direct comparison of conditions with distinct feedback provision within the same task.

Similar to our previous experiments,^21^ participants completed the task twice on different days: once with an error-feedback condition (where they observed the cue ball rolling but did not see the outcome of pocketing) and once with a binary reward-feedback condition (through artificially generated successful trajectories on rewarded trials and no feedback on non-rewarded trials). In the binary reward-feedback condition, participants have no exposure to the real ball trajectories of their shots and hence no error information to learn from. We hypothesized that by eliminating specific visual information in the EVR setting that facilitates error-based learning (i.e., the trajectory of the balls) and reward-based learning (i.e., the target ball pocketing), participants would rely exclusively on the relevant learning mechanism, leading to observable differences in their behavior and neural patterns during training.

Reward-dependent motor variability,^22^ the difference in trial-to-trial change after success vs. failure, was used as the behavioral signature of reward-based reinforcement learning. At the neural level, we focus on post-movement beta rebound (PMBR), a transient increase in beta-band power (12–30 Hz) over sensorimotor cortex following movement cessation.^23^^,^^24^ PMBR amplitude is thought to reflect GABAergic inhibition in the sensorimotor cortex^25^ and has been shown to dissociate the two learning modes: it *increases* across learning during error-based adaptation and *decreases* during reward-based learning.^26^^,^^27^^,^^28^

With the gradual perturbation, unlike previously published abrupt-perturbation,^21^ we expected participants would learn the full rotation in both feedback conditions. We used these behavioral and neural measures to assess the effect of the feedback manipulation, with the predictions that trial-to-trial change after failure would be larger in the reward-feedback than in the error-feedback condition, and that PMBR would decrease across the rotation phase in the reward condition and increase in the error condition. This would be consistent with a pure error- vs. reward-based learning signature. Our results suggest that with a gradual perturbation, visual feedback manipulation can force participants to rely mainly on one learning mechanism.

## Results

### Experimental paradigm

**Figure 1:**
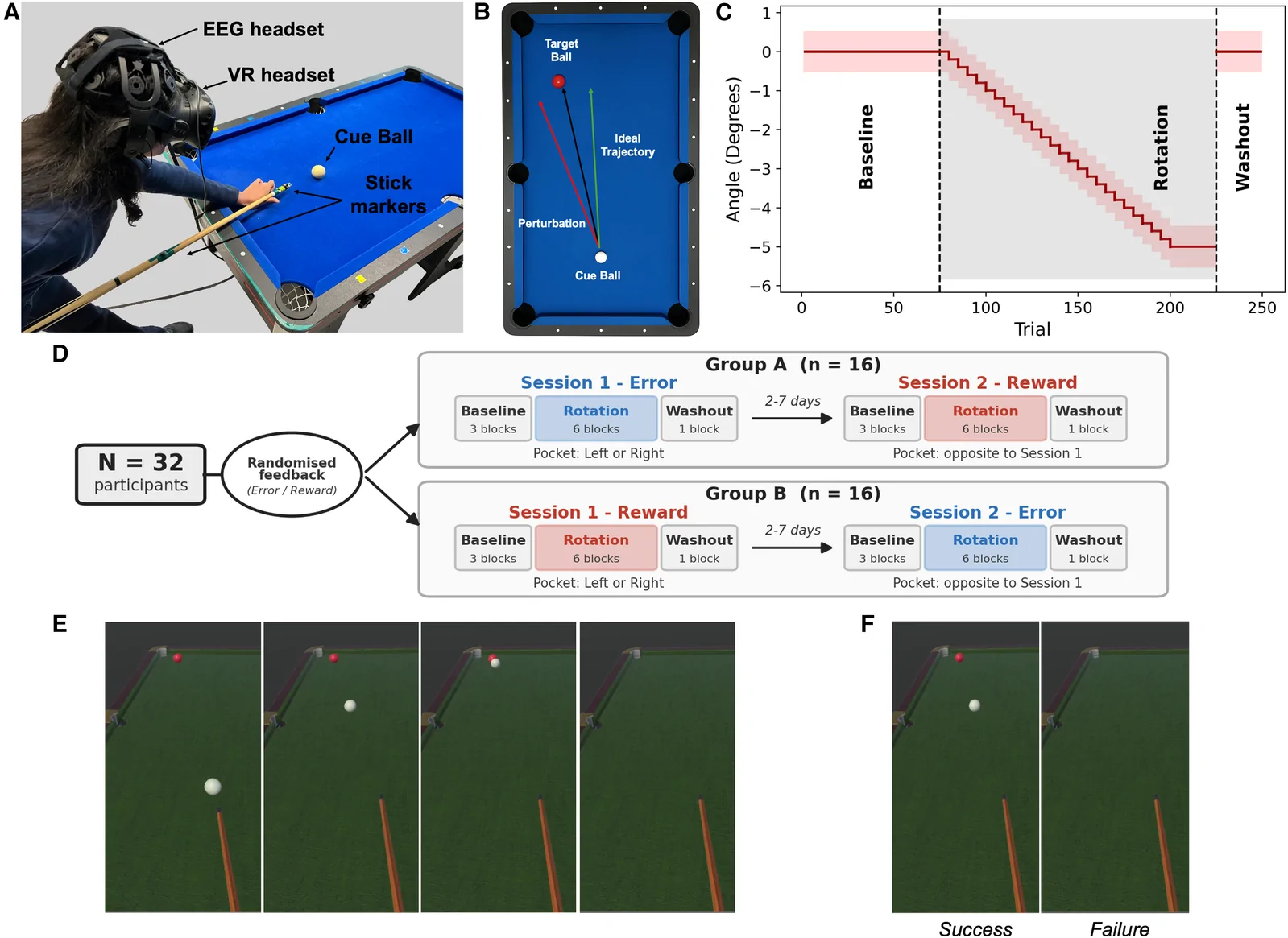
Experimental paradigm (A) Participant playing in the EVR setup (the EEG headset, VR headset, cue ball, and stick markers are labeled). (B) Perturbation applied during the *rotation* phase: the visual trajectory of the cue ball is rotated 5° toward the target pocket side of the table. The figure is shown in the same orientation as (A) (player at the bottom, target pocket at the top). The green line (*ideal trajectory*) indicates the cue-ball direction required to pocket the target ball, while the red line (*perturbation*) indicates the rotated visual feedback shown to the participant under the visuomotor rotation; the black line indicates the cue ball’s actual trajectory toward the target ball. (C) Experimental structure: after a *baseline* phase of 75 trials with full feedback and no perturbation, a visuomotor rotation is applied gradually on the cue ball trajectory (*rotation*) for 150 trials; follows a final phase of 25 trials without perturbation and full feedback (*washout*). The gray shaded region marks the trial range over which the visuomotor rotation is active (the gradual *rotation* phase, blocks 4–9); outside this region (*baseline*, *washout*) the visual feedback is veridical. The solid red line represents the ideal angle of the cue ball trajectory, ramping with the perturbation. The pink area represents the zone that rewards the participant when the shot is directed in any part of the area, which has a total angular width of 1.5° in the reward condition (expanded relative to the natural pocketing range to make reward-based learning feasible without trajectory feedback) and tracks the perturbation throughout the *rotation* phase. (D) Experimental design: the *N* = 32 participants were split, by randomization of the first-session feedback, into two counterbalanced groups of 16. Each participant completed two sessions held 2–7 days apart, one with error feedback and one with reward feedback, shooting toward opposite pockets; the order of feedback condition and of pocket was randomized and counterbalanced across participants. Each session followed the same structure shown in (C), with 25 trials per block. (E) Error feedback (*rotation* phase): the participant sees the cue ball roll along its real trajectory up to the collision (shown as a sequence of frames), providing the sensory prediction error, while the pocketing outcome of the target ball is withheld. (F) Reward feedback (*rotation* phase): the balls are hidden the moment the cue ball is struck; on rewarded (*success*) trials an artificial successful trajectory is displayed, whereas on non-rewarded (*failure*) trials no trajectory is shown, so only the binary outcome is available. No statistical comparisons are shown in this figure.

In this study, *N* = 32 healthy individuals (age 20–46, 11 female, 21 male) participated in a pool game using physical objects while immersed in a VR environment that provided customized visual feedback (Figure 1A). During each trial, participants attempted a single cut shot from a fixed position, aiming to pocket a target ball located near the far left or right corner of the pool table, with no time limit. The experiment was divided into two sessions, held 2 to 7 days apart. Coherently with,^21^ each session included a *baseline* phase (3 blocks of 25 trials), a *rotation* phase (6 blocks of 25 trials), and a final *Washout* block (25 trials).

In the *rotation* phase, a visuomotor rotation was applied gradually on the cue ball trajectory, rotating by 0.2° toward the side of the table every 5 trials (Figures 1B and 1C); to correctly pocket, participants needed to compensate their shot of the same magnitude in the opposite direction.

During each session’s *rotation* phase, participants received either error-only or reward-only feedback via the VR headset. In the error condition, the balls vanished upon collision, whereas in the reward condition, successful shots were reinforced with a fixed successful trajectory, while unsuccessful attempts provided no visual feedback.

To make the reward condition feasible and sufficiently challenging, the range of rewarded angles was expanded to a total size of 1.5° (around the “ideal” direction), based on the results of a pilot experiment that tested different funnel sizes.

All participants took part in two sessions under different conditions, one with error feedback and the other with reward feedback, shooting toward opposite pockets. To prevent order effects, the order of conditions and pockets was randomized and counterbalanced across participants.

To understand the similarities and differences between visuomotor rotation paradigms, the error condition from the previous experiment^21^ was included in this analysis. In that experiment, 32 participants received error-based feedback to correct for an abrupt perturbation of 5°. All other design features remained consistent with the current experiment.

### Task performance across feedback conditions and perturbation schedules

**Figure 2:**
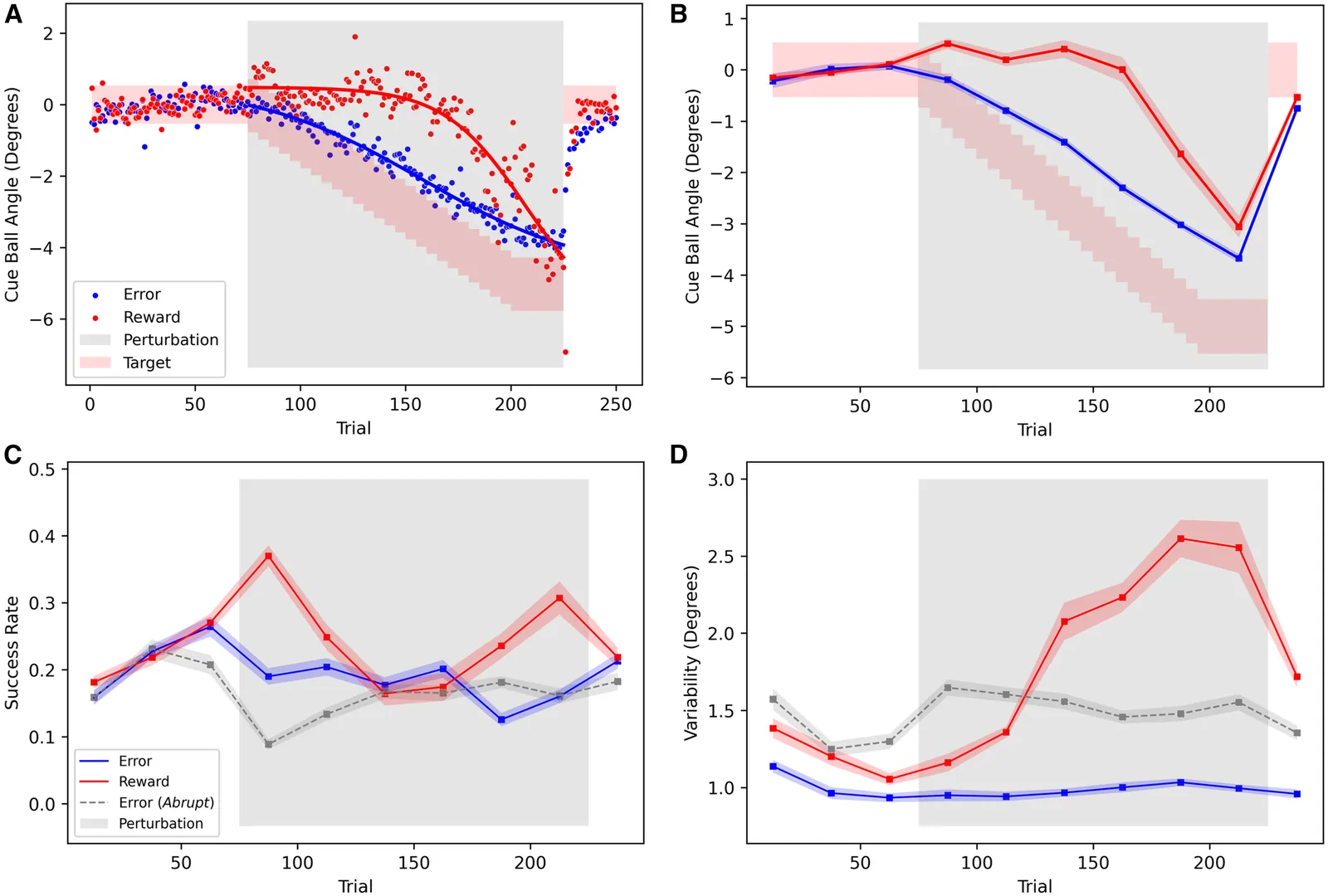
Task performance across feedback conditions and perturbation schedules Task performance in *error* (blue), *reward* (red) and *error abrupt* (gray) conditions. (A) Average cue ball trial-by-trial angle over the participants. The pink area indicates the range of successful angles. The bold line is a logistic fit for both conditions. (B) Average cue ball angle over blocks. (C) Success rate for the three conditions. (D) Corrected variability, after removal of linear trends. (B–D) Each block of 25 trials is represented by its distribution, the means over participants are connected by the bold lines, and the shaded areas represent inter-participant variability (standard error of the mean). The gray area represents the presence of perturbation. *N* = 32 participants for the gradual error and gradual reward conditions (within-participant comparison) and *N* = 32 independent participants for the abrupt error condition. Statistics reported in the text for this figure are repeated-measures ANOVA (gradual error vs. gradual reward), mixed ANOVA (gradual vs. abrupt), Wilcoxon signed-rank tests (paired, within-participant) and Mann-Whitney *U* tests (between cohorts); see the quantification and statistical analysis section. No significance asterisks are displayed in this figure.

In both conditions of the gradual paradigm, participants ultimately compensated for the entire rotation, achieving a comparable magnitude of learning in the last perturbation block (*W*_*Block*9_ = 218, *p* = 0.40; Figures 2A and 2B). Comparing the final compensation to the abrupt error task, the gradual reward condition reached an indistinguishable level of learning (Mann-Whitney *U* = 638, *p* = 0.09), whereas the gradual error condition reached a marginally lower compensation (*U* = 682, *p* = 0.02); this small (∼0.4°) difference reflects the gradual schedule reaching full perturbation magnitude only at the end of the ramp. However, a difference in the rate of learning remained significant (*F*_mode|pert_ (1, 31) = 30.69, *p* < 0.001, *F*_*int*|pert_ (5, 155) = 4.76, *p* < 0.001), as indicated by the parameters of the learning curve fits, reflecting the ongoing influence of feedback provision. Indeed, while the perturbation trend was linear, both conditions were best described by logistic curves, and they exhibited notably different magnitudes (*k*_*error*_ = 0.026, *k*_*reward*_ = 0.061).

Success rates exhibited different patterns (Figure 2C) due to our rewarding scheme. Participants in the *error* condition received consistent information that overlapped with the baseline phase, resulting in a relatively constant rate. Conversely, during the initial rotation phase of the *reward* condition, the task was initially more rewarding due to the expanded reward zone, which made it easier to pocket in the same direction as before. However, as the blocks progressed, the task became more challenging, leading participants to improve their game toward the end. While success rates were similar between conditions during *baseline* (*F*_mode|base_ (1, 31) = 0.15, *p* = 0.70, *F*_*int*|base_ (2, 62) = 2.19, *p* = 0.12), they differed significantly during the *rotation* phase (*F*_mode|pert_ (1, 31) = 4.47,*p* = 0.04, *F*_*int*|pert_ (5, 155) = 6.12, *p* < 0.001). Finally, during washout, they became again similar (*W*_*Block*10_ = 199, *p* = 0.94). On the other hand, while comparing the *error* condition between the *abrupt* and *gradual* tasks, different trends can be observed in the success rate (*F*_mode|pert_ (1, 31) = 2.14, *p* = 0.15, *F*_*int*|pert_ (5, 155) = 8.43, *p* < 0.001), due to a larger drop at the start of the abrupt rotation. This difference can be explained by the rapid change of direction required following the abrupt perturbation, but not the gradual.

Finally, the intertrial variability trends between the error and reward conditions differed significantly due to the information participants used for correction. The reward-based feedback encouraged exploration to discover the correct aiming trajectory, whereas the error condition facilitated a consistent correction across *rotation* blocks with minimal exploration (*F*_mode|pert_ (1, 31) = 65.40, *p* < 0.001, *F*_*int*|pert_ (5, 155) = 10.45, *p* < 0.001). When comparing between the *abrupt* and *gradual* error conditions, similar trends were observed, except for an increase in variability at the start of the *abrupt* rotation phase, resulting from the sudden change in direction needed to correctly pocket the target ball (*F*_mode|pert_ (1, 31) = 40.38, *p* < 0.001, *F*_*int*|pert_ (5, 155) = 2.11, *p* = 0.07).

### Trial-to-trial angle change after success and failure unravels the contributions of the error and reward learning mechanisms

**Figure 3:**
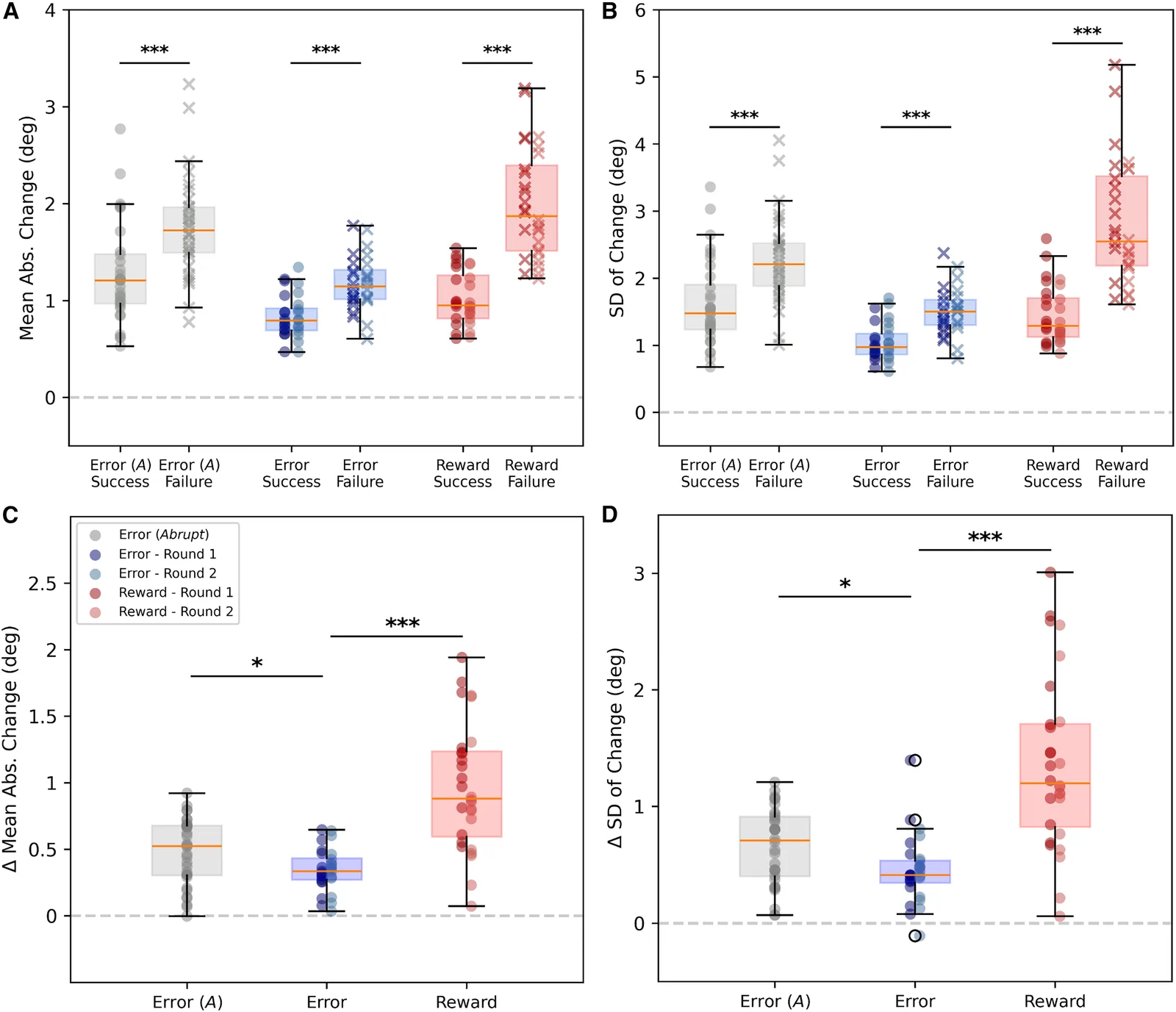
Trial-to-trial angle change after success and failure (A and B) Comparison within condition of the quantities for *error* condition, after success (blue dots) and failure (blue crosses); *reward* condition, after success (red dots) and failure (red crosses); and *error abrupt*, after success (gray dots) and failure (gray crosses). (C and D) Difference (Δ) between condition of changes after failures and successes for the *error* and *reward* conditions of the gradual task, and the *abrupt error* task. The Δ is calculated by subtracting the average change after success and after failure trials, for each participant. All quantities are expressed in degrees. The darker dots represent *Round 1*, while the lighter *Round 2*, for both error and reward. For the *abrupt error* task, both rounds are reported together. The images represent the mean absolute change (A–C) and the standard deviation of change (B–D) for the three conditions. *N* = 32 participants for the gradual error and gradual reward conditions and *N* = 32 independent participants for the abrupt error condition. Within-condition comparisons of change after success versus after failure (A and B) are two-sided Wilcoxon signed-rank tests on paired per-participant values; between-condition comparisons (C and D) are two-sided Wilcoxon signed-rank tests for gradual error versus gradual reward (paired, within participant) and two-sided Mann-Whitney *U* tests for gradual versus abrupt error (independent cohorts). Holm-Bonferroni correction was applied within each of these two test families. Exact test statistics and *p* values are reported in the results text. ∗:*p* < 0.05, ∗∗:*p* < 0.01, ∗∗∗:*p* < 0.001.

To evaluate the contributions of reward-based reinforcement learning and error-based adaptation under the two feedback conditions, we examined metrics associated with reward and error mechanisms. While some measures previously used by us and others in lab-based and real-world tasks, like the inter-trial variability decay,^16^^,^^21^^,^^29^^,^^30^^,^^31^^,^^32^ do not generalize well to gradual learning, others still hold. Specifically, the reward-dependent motor variability—comparison of changes in performance after a success or a failure, linked to reinforcement reward-based learning as it reflects exploration after failures.^22^ The reward-dependent motor variability within the error and reward feedback conditions (Figures 3A and 3B) showed significant differences in how participants adjusted their shooting angle after successful versus unsuccessful shots. In both error and reward conditions, the mean absolute change and variability of change increased after failed shots (*W*_*μ*|*Err*_ = 0, *p* < 0.001; *W*_*σ*|*Err*_ = 2, *p* < 0.001; *W*_*μ*|*Rew*_ = 0, *p* < 0.001; *W*_*σ*|*Rew*_ = 0, *p* < 0.001). That is, as anticipated, participants made greater corrections after a failed shot than after a successful one. This difference between corrections after success and failure, was found in the *abrupt* task as well (*W*_*μ*|*Abrupt*_ = 1, *p* < 0.001; *W*_*σ*|*Abrupt*_ = 0, *p* < 0.001). The raw trial-by-trial angle traces for the abrupt error condition are reported in a previous study.^21^

More importantly, the comparisons between conditions revealed significant differences stemming from the distinct feedback provided (Figures 3C and 3D). The reward condition resulted in a greater increase in variability and more substantial adjustments following failures compared to the error condition (*W*_*Mode*|*μ*_ = 6, *p* < 0.001; *W*_*Mode*|*σ*_ = 5, *p* < 0.001), suggesting that participants explored more following a failure when receiving reward feedback relative to error feedback. When comparing the gradual and abrupt error tasks, a significantly lower change in delta was observed in the gradual task ($U_{Pert\_Mode|\mu }=696,p=0.014$; $U_{Pert\_Mode|\sigma }=703,p=0.011$), highlighting a more pure error-based learning in the gradual task in contrast to the mixture of learning mechanisms in the abrupt error task.

### EEG signature for learning mechanisms: Post-movement beta rebound

While playing pool in VR, participants had their brain activity monitored with wireless EEG to examine trends in PMBR throughout the learning process in the different feedback conditions. PMBR is a well-established marker of sensorimotor cortex activation and it consists of a peak in beta activity (12–30 Hz) following movement cessation. It is associated with motor learning, and its trend is expected to depend on the underlying learning mechanism, increasing and decreasing over the learning, respectively, for error-based learning and reward-based learning.

**Figure 4:**
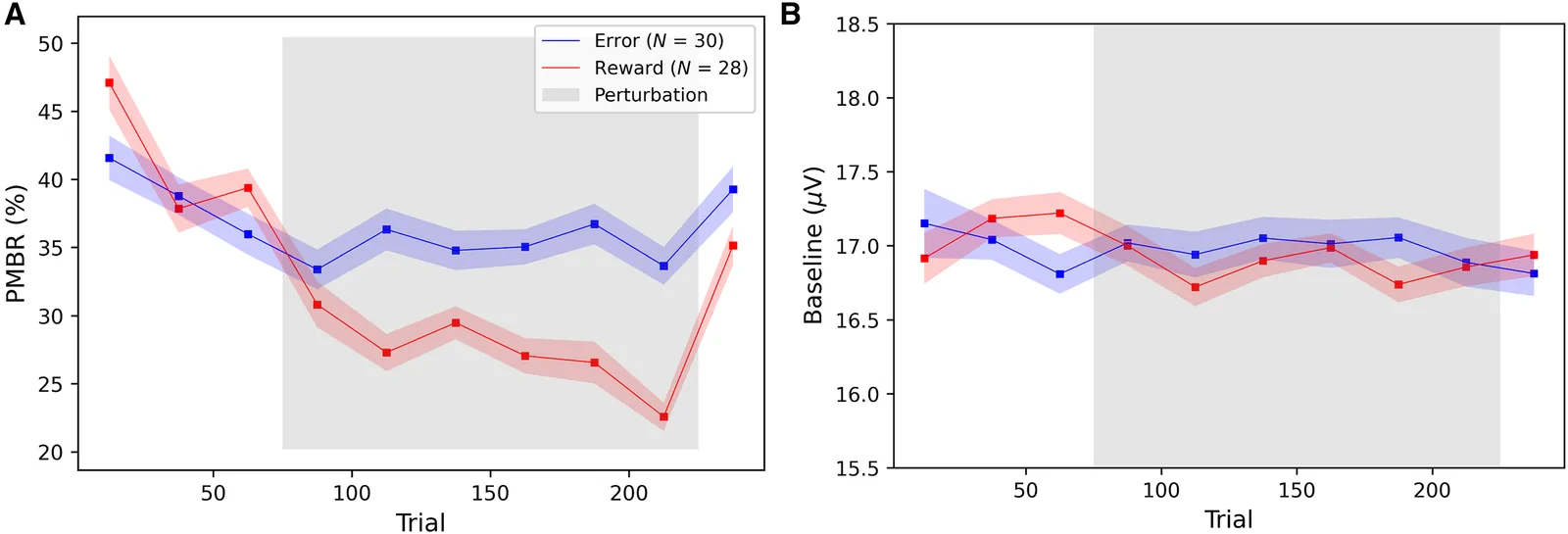
PMBR across learning (A) Average *PMBR* (%); (B) average baseline power for the PMBR calculation. All quantities are calculated within blocks of 25 trials in *error* (blue), *reward* (red) and *error abrupt* (gray) conditions. The shaded areas represent the participants’ variability (standard error of the mean), and the gray area represents the presence of perturbation. After the EEG inclusion criteria described in the method details, *N* = 30 participants contributed to the error condition and *N* = 28 to the reward condition. Trends and between-condition differences were assessed with linear mixed-effects models with random intercepts per participant (348 block-level observations); regression coefficients, *p* values and 95% confidence intervals are reported in the results text. No significance asterisks are displayed in this figure.

When examining the participant data (Figure 4A), both the error and reward conditions displayed comparable PMBR trends during the baseline period (*β*_*Mode*|*Base*_ = 5.28, *p* = 0.31, 95% CI: [–4.97 15.53]; *β*_*Mode*·*Time*|*Base*_ = −1.06, *p* = 0.62, 95% CI: [–5.21 3.08]), characterized by a consistent decline over time.

However, a notable divergence in the PMBR trend emerged during the rotation phase. While participants in the reward condition exhibited a steadily declining behavior (*b*_*Time*|*Pert*_ = −1.31, *p* = 0.008, 95% CI: [–2.28, −0.34]), consistent with past studies on neural trends of reward-based learning, those in the error condition displayed a stable trend throughout the course of learning (*b*_*Time*|*Pert*_ = 0.08, *p* = 0.85, 95% CI: [–0.73, 0.89]). When comparing the two conditions after the perturbation was applied, significant differences emerged (*β*_*Mode*|*Pert*_ = 1.48, *p* = 0.77, 95% CI: [-8.27, 11.22]; *β*_*Mode*·*Time*|*Pert*_ = −1.39, *p* = 0.03, 95% CI: [-2.65, −0.13]), representing the fact that the two feedback mechanisms solicited different learning processes.

No significant differences in baseline beta in the *rotation* phase were observed between feedback conditions (*β*_*Mode*|*Pert*_ = −0.17, *p* = 0.60, 95% CI: [-0.79, 0.46]) or in trends related to the experimental phase (*β*_*Mode*·*Time*|*Pert*_ = −0.006, *p* = 0.87, 95% CI: [-0.09, 0.07]) which confirms that changes in PMBR were not due to underlying beta variations (Figure 4B).

To verify that the divergent PMBR trends were not driven by the difference in absolute success rate between conditions, we tested whether block-wise PMBR tracked block-wise success rate within the rotation phase. Per-participant Pearson correlations between block-wise PMBR and success rate were not different from zero in either condition (error: $\bar{r}=-0.09$, *t*_29_ = −1.07, *p* = 0.29; reward: $\bar{r}=+0.08$, *t*_27_ = +0.87, *p* = 0.39). A mixed-effects model PMBR ∼ SuccessRate with random intercept per participant gave a non-significant slope in both conditions (error: *β* = −7.82, *p* = 0.34; reward: *β* = +1.00, *p* = 0.83), and a non-significant SuccessRate ×Mode interaction in the combined model (*β* = −4.31, *p* = 0.65). The PMBR slope difference between conditions therefore dissociates from task difficulty.

## Discussion

While motor learning is a combination of error-based and reward-based learning mechanisms, isolating those mechanisms in research is useful for improving our understanding of them. Studies in simplified lab-based tasks commonly isolate them via feedback manipulations; yet, in real-world tasks, this is not as simple since the context and complexity of the task allow learning from reward even in the absence of reward feedback.^21^ In this study, we used gradual visuomotor perturbation to keep errors small in an attempt to better isolate error-based and reward-based learning mechanisms in a real-world task. By gradually manipulating the visual feedback during a pool task in an EVR, we isolated those learning mechanisms’ contributions in correcting for the perturbation. Our findings reveal distinct patterns of motor learning and neural activity associated with each feedback type, suggesting that while correcting for small errors, visual feedback by itself can elucidate the complex interplay between error-based and reward-based mechanisms, even within a real-world context.

Our gradual perturbation paradigm allowed the same ultimate level of learning across feedback conditions while exposing clear differences in how participants reached that solution in terms of success rate and inter-trial variability. When visual information about pocketing outcomes was withheld, learners corrected shot direction smoothly and with little exploration, whereas the binary-reward schedule encouraged larger trial-to-trial adjustments and a sustained rise in variability throughout the *rotation* phase.

One key finding was the differential impact of feedback type on behavioral measure, specifically inter-trial variability and trial-to-trial change. In the reward condition, participants exhibited increased variability throughout the learning phase and bigger corrections after failures, which suggests a process of exploration aimed at discovering optimal aiming trajectories. This aligns with reinforcement learning theories, which posit that reward signals encourage exploration to maximize future gains.^33^^,^^34^

Comparing the error conditions with the gradual perturbation studied here and the previously studied abrupt perturbation,^21^ we observed multiple differences in behavior, some are simply due to the different perturbation presented, and some suggest differences in the learning mechanism used. The trends over blocks of success rate and inter-trial variability were very different. In the abrupt task there was a large drop in success rate at the beginning of the rotation phase, followed by an increase in the success rate over blocks. In contrast, in the gradual task, there was a slow trend of decrease in the success rate over learning, as the perturbation gets bigger. This difference can be explained by the difference in the perturbation scheme. The inter-trial variability also had a large change at the beginning of the rotation phase in the abrupt task (an increase), which was not evident in the gradual task and is explained by the difference in the perturbation scheme. Yet, the variability stayed high across all rotation blocks in the abrupt task, leading to a big difference in variability even late into learning, after the errors and success rates are similar between the tasks. This difference in variability cannot be explained by the perturbation schemes and is probably related to the learning mechanisms used, as revealed by the reward-dependent motor variability.

The gradual perturbation task showed very clear differences in the reward-dependent motor variability^22^ between the error-based and reward-based feedback conditions, suggesting a relatively pure error-based learning in the error feedback condition. This is in contrast to the abrupt task, where the error feedback condition showed “reward-like” learning. The reward-dependent motor variability results highlight the better isolation of learning mechanisms in the case of gradual perturbation.

Our neural data analysis of PMBR supported the behavioral findings. It revealed a clear and significant divergence in neural trends between the error and reward conditions during the rotation phase of the gradual task. In the reward-feedback condition, participants exhibited a steadily declining PMBR, consistent with previous studies on reward-based learning,^24^^,^^28^ and with the reward-feedback condition of the previous experiment with abrupt perturbation.^21^ In the error-feedback condition, the participants’ PMBR showed a mild increasing trend, though, not consistent or significant. These findings suggest that even in the context of real-world tasks, error and reward feedback might engage distinct neural circuits and modulate sensorimotor cortex activity differently during motor learning. Importantly, the results of the experiment with abrupt perturbation^21^ suggested that when experienced errors are large, the correction for the error itself is rewarding enough to engage the reward-learning mechanism even in the absence of a reward signal. Here, we show that when the errors are small (as a result of gradual and small perturbation changes), one can suppress the reward learning mechanism and isolate the neurobehavior responses of a relatively pure error-learning mechanism.

In conclusion, this study reveals distinct patterns of motor learning and neural activity associated with error-only and reward-only feedback in a real-world motor learning task, delivered via EVR. This, presumably, isolates error-based and reward-based learning mechanisms. Our results highlight the complex interplay between error and reward processes in motor skill acquisition, which can be isolated only in the case of small errors provided in a gradual learning paradigm. This suggests that gradual perturbation schedules may offer a relatively pure error-based learning, even in a more ecological real-world task. These findings provide not only an approach to studying error-based learning in real-world task, but also an approach for neurorehabilitation and skilled motor learning, which can optimize error-based mechanisms. Accordingly, this has implications for understanding motor skill learning and rehabilitation, as well as for the development of more effective training interventions by leveraging the unique capabilities of EVR.

### Limitations of the study

While this study offers valuable insights into real-world motor learning, it is important to acknowledge some limitations. First, the fidelity of our mobile EEG recordings is constrained by motion-related artifacts, as muscle activity and movement can contaminate beta-band activity, and ICA pipelines might not be enough to eliminate such artifacts during dynamic tasks.^35^ Second, because all participants were healthy young adults, additional studies with older adults would be needed for rehabilitation purposes, as older individuals adapt more slowly, rely less on explicit strategies, and exhibit altered reward-based modulation of retention, suggesting that the adaptation-reinforcement balance may shift when dopaminergic signaling declines with age or in neurological populations.^36^^,^^37^ Third, the two sessions were spaced over a relatively broad window (2–7 days); although the realized intervals were tightly distributed (mean 4.25 days, SD 1.56) and balanced across condition orders (Mann-Whitney *p* = 0.58), so that the spacing is unlikely to have systematically biased the between-condition comparison, we cannot fully exclude an effect of inter-session interval on retention.

## Resource availability

### Lead contact

Requests for further information and resources should be directed to and will be fulfilled by the lead contact, Shlomi Haar (s.haar@imperial.ac.uk).

### Materials availability

This study did not generate new unique reagents or materials.

### Data and code availability

• All behavioral and EEG data reported in this study have been deposited at FigShare and are publicly available: https://doi.org/10.6084/m9.figshare.31228423.
• All original code has been deposited at FigShare and is publicly available: https://doi.org/10.6084/m9.figshare.31228423.
• Any additional information required to reanalyze the data reported in this study is available from the lead contact upon request.

## Acknowledgments

F.N. is supported by 10.13039/100014013UK Research and Innovation (UKRI Centre for Doctoral Training in AI for Healthcare grant number EP/S023283/1); A.A.F. acknowledges a UKRI Turing AI Fellowship grant (EP/V025449/1); S.H. was supported by the Edmond and Lily Safra Fellowship Program and by the UK Dementia Research Institute Care Research & Technology Centre.

## Author contributions

S.H. and A.A.F. conceived and designed the study; F.N., A.A.F., and S.H. developed the experimental setup; F.N. and A.A. acquired the data; F.N., A.A.F., and S.H. analyzed and interpreted the data; F.N. drafted the manuscript; S.H. and A.A.F. revised the manuscript.

## Declaration of interests

The authors declare no competing interests.

## STAR★Methods

### Key resources table

**Table:** 

REAGENT or RESOURCE	SOURCE	IDENTIFIER
**Deposited data**		

Behavioral data (cue ball trajectories, shot angles, trial outcomes), *N* = 32 participants × 2 sessions	This paper	FigShare: https://doi.org/10.6084/m9.figshare.31228423
Pre-processed EEG data and block-level PMBR values	This paper	FigShare: https://doi.org/10.6084/m9.figshare.31228423
Original analysis code (behavioral analysis, EEG pre-processing, time-frequency decomposition, statistics)	This paper	FigShare: https://doi.org/10.6084/m9.figshare.31228423
Abrupt-perturbation dataset (error condition), used here for comparison	Nardi et al.^19^^,^^20^^,^^21^	See “data and code availability” statement in the cited paper

**Software and algorithms**		

Unity (Embodied Virtual Reality environment, C#)	Unity Technologies	https://unity.com
MATLAB (EEG pre-processing and time-frequency analysis)	MathWorks	RRID:SCR_001622
EEGLAB v2022.1	Delorme and Makeig^38^	RRID:SCR_007292
FieldTrip	Oostenveld et al.^39^	RRID:SCR_004849
clean_rawdata/Artifact Subspace Reconstruction	Kothe and Jung et al.^40^	https://github.com/sccn/clean_rawdata
ICLabel	Pion-Tonachini et al.^41^	https://github.com/sccn/ICLabel
Python (behavioral analysis and statistics)	Python Software Foundation	RRID:SCR_008394
SciPy	Virtanen et al.^42^	RRID:SCR_008058
statsmodels (linear mixed-effects models)	Seabold and Perktold^43^	RRID:SCR_016074
pingouin (ANOVA, non-parametric tests)	Vallat^44^	https://pingouin-stats.org
NumPy	Harris et al.^45^	RRID:SCR_008633
pandas	McKinney^46^	RRID:SCR_018214
Matplotlib	Barrett et al.^47^	RRID:SCR_008624

**Other**		

DSI-7 wireless dry-electrode EEG headset (7 channels, 300 Hz)	Wearable Sensing	https://wearablesensing.com
HTC Vive virtual reality headset	HTC Corporation	https://www.vive.com
OptiTrack motion capture cameras (4 units)	NaturalPoint	https://optitrack.com
Regulation pool table, cue stick and cue ball with reflective markers	N/A	N/A

### Experimental model and study participant details

Thirty-two healthy human volunteers (11 female, 21 male; age 20–46, mean 25.9 ± 6.2 years) took part in the study. All participants were right-handed, had normal or corrected-to-normal vision, and had minimal previous pool experience. Ethnicity was not recorded. Participants were recruited from the staff and student population of Imperial College London and the surrounding community, and received no financial compensation.

Sex was self-reported at recruitment. Participants were recruited irrespective of sex, and sex was not used to stratify the randomisation of feedback condition or target pocket. The study was not powered to detect effects of sex or gender, and sex was not included as a factor in any analysis, so we report no influence of sex or gender on the behavioral or neural measures; all results are pooled across sexes. As ethnicity was not recorded, no analysis of race or ethnicity was performed.

All experimental procedures were approved by the Imperial College Research Ethics Committee (ICREC; protocol number 21IC6754) and were performed in accordance with the Declaration of Helsinki and with all relevant institutional and national regulatory standards for research involving human participants. All participants gave their written informed consent before participating in the study.

### Method details

#### Experimental setup

In our experimental paradigm participants engage in a physical pool game while wearing a VR headset, receiving customised visual feedback within the virtual environment, which is specifically designed to target particular learning mechanisms. The Embodied Virtual Reality (EVR) setup, developed using Unity software programmed in C#, integrates a physical regular pool table with Virtual Reality to precisely measure and manipulate participants’ actions. To enhance the sense of realism, pre-experimental calibration, utilising cameras and VR controllers positioned on the pool table, ensures alignment between the dimensions of the virtual and real tables. We incorporate as well a 7-channel wireless electroencephalogram (EEG) device, to record brain activity during experiments, which can be used in conjunction with the HTC Vive headset without causing any interference.

The embodiment allowed by our setup facilitates physical interaction with real-world objects, providing comprehensive somatosensory and proprioceptive feedback, as the physical cue stick is replicated in VR, with its geometric characteristics and marker positions streamed via four high-precision Optitrack cameras, allowing participants to perceive it as a virtual object. Preserving haptic feedback and realistic collision sounds, participants strike a physical cue ball. However, collisions between the cue ball and target balls are represented by artificial sounds, as the target balls are entirely virtual but programmed to adhere to real-world physics, similar to other objects within the virtual environment.^18^

#### Experimental design

The sample size was chosen to match our previous within-subject EVR study,^21^ which used the same setup and analysis pipeline. A post-hoc sensitivity analysis (paired test, *α* = 0.05 two-tailed, powe = 0.80) gives a minimum detectable Cohen’s *d*_*z*_ of 0.51 at *N* = 32 for the within-subject behavioral comparisons; for the between-subject comparisons against the abrupt cohort the minimum detectable Cohen’s *d* is 0.71–0.74. The block-level PMBR analyses use a mixed-effects model with random intercepts per participant (348 observations), which has greater sensitivity than a per-subject paired test and tolerates the asymmetric per-condition retention rates after EEG cleaning.

Coherently with our previous studies,^20^^,^^21^ each session comprised 3 blocks of 25 shots for the *Baseline* phase, 6 blocks for the *Rotation* phase, and a final *Washout* block. During each trial, participants executed a single cut shot, aiming for a target ball positioned near the far-left or far-right corner of the pool table, initiating from a fixed location without time constraints. All participants engaged in two sessions under varying conditions, one involving error feedback and the other involving reward feedback, shooting toward opposing pockets. The two sessions were held 48 h to 1 week apart, ensuring that they were not too close to avoid immediate recall of the previous session nor too far to retain some of the learning they experienced in the first session. The realised inter-session interval (extracted from the recording timestamps of the Block-1 EDF files) was 4.25 days on average (median 4.11, SD 1.56) and did not differ between condition orders (Error-first: mean 4.13 days, SD 1.73; Reward-first: mean 4.38 days, SD 1.42; Mann–Whitney *U* = 113, *p* = 0.58). The sequence of tasks and pocket selections were randomised and counterbalanced both between and within participants.

During the *Rotation* phase, a visuomotor rotation was applied to the cue ball’s trajectory, necessitating that participants adjust their shot direction toward the center of the table to successfully pocket the ball. Expanding on our previous experiment and addressing the limitation of imbalanced learning between the two conditions, we implemented a gradual perturbation paradigm. In this paradigm, the magnitude of the rotation was incrementally increased until it reached the target size of 5°. Specifically, starting from the fourth block, the shot direction was rotated by 0.2° every 5 trials, ensuring that the transition was gradual and barely perceivable by the participants. This paradigm also ensured that the final rotation block had a constant perturbation magnitude on its full size, which was used to measure learning.

Specific feedback related to the condition was provided through virtual reality in the *Rotation* blocks. The task was structured to force participants to rely on a particular learning mechanism by removing the visual information related to the opposite learning mechanism.

In the error-based feedback condition, participants were shown only the cue ball’s trajectory up to the point of collision. This forced them to depend on errors in the cue ball path to learn, as they could not see if the target ball was successfully pocketed. In contrast, the reward feedback condition involved concealing the balls the moment the cue ball was hit. Successful shots were positively reinforced by showing participants a simulated successful trajectory, while unsuccessful attempts resulted in no trajectory being displayed.

The criteria for reward allocation were established by initially identifying a range of angles typically leading to successful pocketing under normal circumstances, and subsequently widening this range to ensure the task remained achievable without extra feedback. Following preliminary experiments, the success range was broadened, leading to a total area of 1.5°. Enlarging the success funnel was necessary to control success rate in the absence of trajectory feedback: with the unexpanded range, the binary reward signal in the rotation phase would have been too sparse to drive learning. The expansion size was chosen as the smallest value that, in the pilots, still allowed reward-based learning to take place. The ideal trajectory was modified to reflect the rotation magnitude throughout the session, so the rewarded zone tracked the perturbation rather than remaining anchored to the unperturbed direction.

#### EEG acquisition and pre-processing

EEG data was recorded using a wireless DSI-7 headset with 7 channels, positioned under the VR headset. The 7 channels follow the international 10–20 system: F3, F4, C3, C4, P3, P4 and Pz, with Pz used as the on-line reference. The data was sampled at 300Hz and synchronised using trial timestamps with the VR data. Pre-processing was conducted in MATLAB, through the EEGlab^48^ and Fieldtrip^39^ toolboxes.

Initially, FIR filters were applied for high-pass filtering at 1Hz and low-pass filtering at 45Hz, due to the frequencies of interest. Subsequently, the EEGlab clean_rawdata function was used to eliminate artifacts from the signals, including noisy data segments and problematic channels, by using the Artifact Subspace Reconstruction algorithm.^40^ Given the channel sparsity, the minimum correlation between channels was set to 0.6 to preserve as much informative data as possible while minimising the removal of channels (at most 1) or retention of non-informative data. Additionally, signal windows were removed based on thresholds for the maximum standard deviation of bursts and the maximum fraction of contaminated channels permitted in the final output data for each considered window. These parameters were selected by examining time-frequency plots of the sessions and evaluating three combinations of parameter settings with progressively stricter criteria, choosing the one that maximised retained participants as well as cleaned the data best. After data cleaning, removed channels were reconstructed through spline interpolation, based on the international 10–20 EEG system.

In the field of motor neuroscience, a notable neural event following voluntary movement is the Post-Movement Beta Rebound (PMBR), characterised by a temporary increase in beta oscillation power across the sensorimotor network after movement cessation.^23^ PMBR, linked to motor learning, is believed to reflect GABAergic activity, with its amplitude varying based on learning mechanisms, increasing during error-based adaptation and decreasing during reward-based learning.^25^^,^^26^^,^^27^^,^^28^ To capture both baseline and post-movement activity, epochs in our study were set to a duration of 9 s around each billiard shot, providing sufficient time to observe fluctuations in beta power. Given that PMBR is typically observed within 1000 ms after movement offset,^24^ our analysis focused on this critical window to identify peak beta activity and assess PMBR dynamics.

Following epoch extraction, Independent Component Analysis (ICA) was performed to remove artifacts related to muscle movement, eye activity, and heart function. ICA decomposition used the extended Infomax algorithm, and components were classified automatically with ICLabel^41^; components with greater than 70% probability of belonging to the Muscle, Eye, Heart or Channel Noise classes were rejected, and the remaining components were used to reconstruct the cleaned signal. Based on prior motor learning studies^26^^,^^49^ and the right-handedness of all participants, only data from the C3 channel was selected for further analysis. Epochs were extracted from the EEG data, centered on the onset of cue ball movement to provide a temporal reference for the participants’ actions, consistent with previous research on Event-Related Potentials and event-related spectral perturbations.^16^^,^^21^

#### EEG time-frequency analysis

Following the preprocessing of the EEG data, a time-frequency analysis was performed to examine the brain activity associated with participant movements during the experiment, concurrent with the provision of task-related feedback. Time-frequency decomposition used complex Morlet wavelets, with a wavelet width of 3 cycles and a Gaussian envelope width of 0.8, evaluated in 1 Hz steps over 8–45 Hz. PMBR was extracted from the 0–1000 ms window post movement offset,^24^ computed as the median across trials of the peak % change in beta-band (12–30 Hz) power within this window relative to the per-frequency baseline. After time-frequency domain transformation, the data were standardised to equate the total power in the 8-45Hz frequency range across all sessions to ensure comparability across sessions. Specifically, the total power was computed to derive the normalisation factors for each session.^50^ This normalisation technique ensures that beta variations are more pronounced and accurate, thereby facilitating precise detection of fluctuations attributable solely to the signal itself, as potential trends and patterns would be consistently observable across all frequencies, not exclusively within the beta band.

Furthermore, the normalisation across sessions mitigates any trends in the baseline, defined as the median power over each session, given that the correction is uniformly applied between the different blocks of the same session.

The following analysis involved the calculation of EEG power change in response to events (cue ball strike) as a percentage change, normalised relative to the median power evaluated in 1Hz steps across each block. To ensure robustness, the peak value was computed on the average signal across the beta-band frequencies, yielding a single value per trial, of which the median was selected to obtain a PMBR value for each block. The median was preferred over the mean because it is robust to single trials with residual motion-related artifacts that survive ICLabel-based component rejection, which are common in mobile EEG during whole-body movement. Additionally, to increase data reliability, criteria were established to selectively include or exclude specific sessions from the task analysis. EEG data may contain missing values, potentially due to electrode misplacement resulting from the substantial movements required by the task. Individual blocks were included into the EEG time-frequency analysis only if at least half of the block contained available data. Entire sessions were included in the analysis if a maximum of 3 blocks (max 2 in the *Rotation* phase) were missing, to accurately capture trends and prevent the inclusion of potentially noisy information. Following the selection of reliable sessions, missing data were imputed separately for error and reward sessions, employing *Probabilistic Principal Component Analysis*.

For the reward and error tasks, 7.79% and 8.18% of the values were imputed, respectively, contingent upon the strict threshold of having at least 7 blocks already present per session. The comprehensive data preprocessing resulted in a reduction in the number of participants included in the EEG analysis from 32 to 28 and 30 for the reward and error tasks, respectively. Finally, all block values were aggregated by block and reported by condition.

### Quantification and statistical analysis

Statistical details for each analysis, including the tests used and the value of *N* and what it represents, are given in the results text and in the corresponding figure legends. Unless stated otherwise, *N* is the number of participants: *N* = 32 for all behavioral analyses of the gradual task (within-participant comparison of the error and reward sessions), *N* = 32 independent participants for the previously published abrupt error cohort,^21^ and *N* = 30 (error) and *N* = 28 (reward) for the EEG analyses after the inclusion criteria described in the method details. All tests were two-sided and significance was defined as *p* < 0.05. Where asterisks are shown in a figure, they denote ∗*p* < 0.05, ∗∗*p* < 0.01, ∗∗∗*p* < 0.001.

Behavioral data were analyzed coherently with,^21^ considering blocks of 25 trials to ensure the observed trends were robust and reliable. Given the absence of significant differences between shots directed toward the left and right pockets, data from both directions were combined to enhance statistical power. Similarly, no differences emerged among participants based on whether they performed the task in their first or second session with the same type of feedback.

The raw behavioral data underwent preprocessing to handle outliers. Specifically, a threshold of 3 standard deviations was determined to eliminate outliers in cue ball directional errors during the baseline and perturbation learning phases. This process resulted in the removal of 1.27% and 3% of the data for the error-based and reward-based tasks, respectively.

To measure learning, the main focus of the behavioral data analysis was the directional error participants made on each shot. After determining the ideal trajectory for a successful shot (from^19^), the cue ball angle was measured by calculating the deviations from this ideal direction.

As the visuomotor rotation was introduced during the rotation phase, the “ideal” angle was adjusted from 0° to match the rotation size, and results are presented accordingly. The data were then fitted to several potential learning curves, with the optimal curve selected based on *F*-tests comparing the residual sum of squares across exponential, double exponential, linear, and logistic models, chosen according to traditional learning models. The parameters for the best-fit curves are detailed in the results section.

The method for determining the success rate varied depending on the phase of the experiment and the feedback condition. In the baseline and washout phases, as well as during the perturbation phase of the error condition, the success rate was calculated as the proportion of successful shots. However, during the reward feedback session, success was determined by whether or not participants were shown a successful trajectory, over the total number of shots.

Shot variability was analyzed to account for inherent uncertainties and to maintain consistency with prior research.^18^^,^^21^ This analysis focused on measuring the corrected variability, which involved calculating the standard deviation of the residuals after removing any linear trends from the directional error in shots at the block level. This approach remained valid despite the presence of an overall logistic trend at the session level, as a sufficiently linear trend was observed within blocks of 25 trials.

To investigate error-like and reward-like learning across the two conditions, the reward-dependent motor variability was employed to ascertain the relationship between these mechanisms and the conditions. The degree to which motor variability relies on reward is determined by assessing changes in performance from trial to trial following success or failure. This metric is typically associated with reinforcement-based motor learning,^22^ where variability increases after unsuccessful trials, suggesting the system is exploring alternative motor strategies to achieve success. To calculate reward-dependent motor variability, the distributions of changes in shot angle after successful and unsuccessful trials were considered for each participant. These are presented in Figure 3 with error bars (the box represents the IQR and the orange line is the median).

The statistical methodology employed repeated-measures and mixed ANOVA, alongside mixed-effect linear models, to analyze the repeated measurements obtained from each participant. Repeated-measures were selected when all participants provided multiple measurements across both sessions, whereas mixed ANOVA was chosen to accommodate differences between conditions. The ANOVA tests reported in the results section pertain to the baseline (*F*_·|base_), perturbation (*F*_·|pert_), or washout phases (*F*_·|wash_) and were conducted to assess differences between modes (*F*_mode|·_) or the interaction between mode and time (*F*_*int*|·_); degrees of freedom are reported in parentheses with each *F* value.

Non-parametric tests, including paired Wilcoxon Signed-Rank tests and Mann-Whitney tests, were used for sample testing and comparisons to address non-normal distributions within the samples. Specifically, Wilcoxon Signed-Rank tests were utilised to compare single samples against a zero median or paired samples, while Mann-Whitney tests were applied to compare different samples. Within the two test families that contain multiple non-independent paired comparisons on the same dependent measure (the within-condition trial-to-trial change tests across error, reward and abrupt-error conditions, and the between-condition trial-to-trial change tests), Holm–Bonferroni correction was applied; all reported effects in these families remained significant after correction. Tests on different dependent measures (learning rate, success rate, inter-trial variability, PMBR slope) were not pooled into a single family, since they address distinct pre-specified hypotheses.

Linear mixed-effects models with a random intercept per participant were used for the block-level PMBR analyses, with regression coefficients reported together with their *p* values and 95% confidence intervals. Behavioral analyses and statistical testing were performed in Python (SciPy, statsmodels and pingouin); EEG pre-processing and time-frequency decomposition were performed in MATLAB using EEGLAB and FieldTrip. Versions of all software packages are listed in the key resources table.
